# Beliefs about Causes and Consequences of Obesity among Women in Two Mexican Cities

**DOI:** 10.3329/jhpn.v30i3.12295

**Published:** 2012-09

**Authors:** Arturo Jiménez-Cruz, Yolanda Martínez de Escobar-Aznar, Octelina Castillo-Ruiz, Raul Gonzalez-Ramirez, Montserrat Bacardí-Gascón

**Affiliations:** ^1^Facultad de Medicina y Psicología, Universidad Autónoma de Baja California, Mexico; ^2^Unidad Académica Multidisciplinaria Reynosa-Aztlán, Universidad Autónoma de Tamaulipas, México; ^3^Colegio de la Frontera Norte, Tijuana, Baja California, Mexico

**Keywords:** Beliefs, Causes, Consequences, Obesity, Treatment, Mexico

## Abstract

Personal beliefs might be barriers to the prevention and treatment of obesity. To assess the beliefs about causes and consequences of and possible solutions to obesity among 18-40 years old women in two Mexican cities and to analyze the association with demographic variables, we developed a questionnaire and assessed the women's weight status. The questionnaire was applied at two outpatient healthcare centres and assessed the responses by the Likert scale. Results were analyzed by demographics, using the chi-square and Spearman correlations. One thousand one hundred adult women participated in the study. Mean age was 27.8 years, and mean BMI (kg/m^2^) was 27.05. The prevalence of overweight and obesity was 35% and 24% respectively. The most mentioned causes of obesity were eating oil and fat (4.1), fried foods (4.1), and eating too much (4.00). The most reported consequences were diseases (4.1), discrimination (3.9), and early death (3.7). The main solutions were physical activity (4.2), healthful eating (4.2), and personal motivation (4.1). Age of participants higher than 30 years, living with a partner, having more than 6 years of education, and having overweight and obesity were predictors of more knowledge about the causes, consequences, and solutions. These Mexican women from low SES had reasonably good knowledge about the causes and consequences of obesity. Although improving education might be beneficial to prevent obesity, changes in environmental contingencies are also necessary to prevent this epidemic.

## INTRODUCTION

Mexico is one of the countries with the highest prevalence of childhood obesity. It has been reported that the possible risk of overweight and obesity among infants and toddlers reaches as far as 42% in some regions (1). Similar results have been found in school-age children (2). In adult populations, young women living in poverty demonstrate prevalence of obesity that triples over a period of 4 to 8 years (3). The magnitude and implications of adult and childhood obesity make preventive strategies a public-health priority. False beliefs or myths about the causes of obesity could be barriers for the adherence to adequate preventive strategies and treatment (4,5). According to Brickman *et al*., behavioural changes are more effective when a learning process has occurred; therefore, it is important to identify the beliefs about causes and consequences of and solutions to a disease (6). In Mexico, we have found no studies exploring the beliefs about causes and consequences of and treatment for obesity; thus, the purpose of this study was to assess beliefs about the causes and consequences of and possible solutions to obesity in a sample of low-income women in two cities that border the USA and Mexico as well as to analyze those beliefs with demographic variables.

## MATERIALS AND METHODS

### Settings

Baja California (known as Baja) is the extreme northwestern Mexican state. Its largest city is Tijuana, and it borders the US state of California. In Mexico, those who are not eligible to receive formal institutional healthcare under social security are eligible to participate in the primary healthcare settings of the Insituto Estatal de Salud (IES), and these patients usually come from the lowest income levels. In Baja, approximately 38.6% of the total population is eligible for the IES healthcare. Tamaulipas is a northeastern Mexican state, and it borders the US state of Texas, and Reynosa is located across the Rio Grande (Río Bravo) from McAllen in Hidalgo County in the US state of Texas. In Reynosa, more than 50% of the total population is eligible for healthcare at the IES (7).

### Subjects

One thousand one hundred women older than 18 years, attending the waiting room in a healthcare centre in Tijuana and Reynosa, participated in the study. Pregnant women and women with physical or mental disabilities were excluded.

### Sets of questionnaire

Three open questions about causes, consequences, and remedies of obesity were used in designing a questionnaire, and it was first applied to college students. A list of the most frequent responses was used in making the second questionnaire. This questionnaire was designed on a 5-point Likert-type scale from “strongly disagree” to “totally/completely agree” (1-5). After administering three sets of questionnaire on a 5-point Likert-type scale, the reliability (r) of the final questionnaire obtained through test-retest applied to 30 individuals was 0.89.

The final questionnaire consisted of 29 items which were divided into four sections: (i) beliefs about the causes, (ii) beliefs about the consequences, (iii) beliefs about solutions, and (iv) weight changes. The Cronbach alpha was 0.88. Demographic data included: age, sex, marital status, education, place of origin, number of years living in Baja California or Tamaulipas, weight, height, and weekly income.

The study was approved by ethics committee of the nutrition academic group of the Universidad Autónoma de Baja California, and the participants signed an informed consent form.

The questionnaire was applied through direct interview in the waiting room. Weight and height were measured using standardized techniques with a scale and a stadiometer.

The sets of questionnaire were applied from March to October 2009.

### Statistical analysis

Cronbach's alpha was used in testing internal validity and the Spearman Rho correlation to measure reproducibility. Results were compared according to sociodemographic variables, and the beliefs about causes, consequences, and treatment, using binomial logistic regression.

## RESULTS

In Table 1, the general characteristics of the population are shown. One thousand one hundred adult women participated in the study. The average age was 37.5 years (range: 18-92). Thirty-five percent were overweight, and 24% were obese; average BMI was 27.1 kg/m^2^; high-school and college graduates were 29% of the sample, and 3% had not completed elementary school. Eighty percent had a monthly income of less than 650 dollar, and 56% were internal immigrants (born in other Mexican states).

Causes with the highest scores (mean Likert scores) were: eating fat (4.09); eating fried foods (4.05); high consumption of foods (4.00); consumption of cakes, chocolates, and sweets (3.99); and consuming soft drinks (3.86). The consequences with the highest score were: diseases (4.08), discrimination (3.90), early death (3.72), anxiety (3.64), and low self-esteem (3.56). The highest-ranking ways of solutions were: increasing physical activity (4.2), healthful eating (4.19), personal motivation (4.07), professional treatment (4.0), and eating at home (3.8).

However, on the extreme of the distribution, it was observed that a high-risk group had misconception about causes and implications of and solutions to obesity: 15% of the population did not consider that a sedentary lifestyle, consuming soft drinks or an unhealthy lifestyle were risk factors for obesity; additionally, 22% did not consider that obesity could cause early death, 39% did not believe that less TV-viewing could help reduce obesity, and 14% thought that liposuction was the right treatment for obesity.

No differences were observed when all the beliefs were compared by migration status, or the knowledge about the causes by level of income. However, people who are over 30 years of age (according to 95% CI=1.33-3.04) and have an educational level above primary education are more likely to believe that spending a lot of time sitting or lying down is a cause of obesity. Additionally, being older than 30 years or living with a spouse, or having more than six years of education and being overweight and obese are factors that make people more likely to agreeing with most of the items in the questionnaire (Table 2).

**Table. 1.** General characteristics of the study population

Variable	Tijuana, Baja California		Reynosa, Tamaulipas	
	No.	%	No.	%
Level of education				
None	19	3	2	1
Some elemmentary	69	12	111	22
Elemmentary	168	28	214	43
Middle	236	39	119	24
High school	108	18	52	10
Civil status				
Single	182	30	74	15
Married	267	45	252	50
Free living (union libre)	151	25	174	35
Number of children				
0	72	12	27	6
1	97	16	171	34
2	121	20	146	29
≥3	310	52	156	31
Body mass index (kg/m^2^)				
<18.5	10	2	12	2
18.5-<25	152	25	219	44
25-<30	206	34	181	36
>30	232	39	88	18
Weekly income				
<$1200	260	43	216	43
$1200-<$2000	219	37	196	39
$2000-<$4000	101	17	69	14
≥$4000	20	3	19	4
Born in the state	185	31	249	50

## DISCUSSION

The results of this study suggest that low-income Mexican women had reasonably good knowledge about the causes and consequences of and treatment for obesity. However, there was a high-risk group with great misconceptions about those issues. These data are consistent with those of a former study conducted among mothers of 6 to 24 months old infants and who did not consider consumption of sweetened drinks and foods with high fat content as risk factors for the development of childhood obesity (2). Several authors have suggested that dietary practices are established early in life, and the type of foods introduced might model the food habits that will continue throughout childhood (8-10). Therefore, since the younger women of this study had misconceptions about the causes and consequences of and treatment for obesity, if pregnant, they will become role models for their children's early feeding practices and lifestyle habits (1). To our knowledge, intervention programmes for low-income young women have received little attention. Thus, this attitude may fail to prevent inadequate lifestyle habits resulting in augmentation of childhood obesity. There is an urgent need for a special focus to promote prevention strategies for this group. On the other hand, being older than 30 years, living with a spouse, having more than six years of education, and being overweight or obese were predictors of better knowledge about the causes and consequences of and treatment for obesity. These results are consistent with those reported by Covic *et al.* (4) in Australian population, and more recently among low-income Latino women and those with a higher level of education (11). Covic *et al.* reported that adults believe high consumptions of fat and sugar are among the main causes of obesity (4). Wang and Coups reported a national probability survey among US adults and showed that most people believe obesity is caused by unhealthy food habits, lack of physical activity, and that genetics plays a minor role (12). Likewise, up to 77% of low-income Latinos identified at least one cardiovascular disease as a consequence of obesity and also believed that obesity started early in life (11), and the most reported treatments for obesity were similar to those found among Australian women (4), which are consistent with beliefs about the causes.

**Table. 2.** Unadjusted odds ratio of being in agreement with the causes and consequences of and treatment for obesity

Area of belief	≥30 years OR (95% CI)	Living with a spouse OR (95% CI)	>6 years of education OR (95% CI)	Income <$ 400/month OR (95% CI)	Being overweight and obese OR (95% CI)
Causes					
Spending a lot of time sitting or lying down	2.01 (1.33-3.04)‡	1.30 (0.93-1.82)	2.92 (2.15-3.96)‡	1.33 (0.93-1.20)	1.80 (1.26-2.55)‡
Eating foods that will make you fat	2.00 (1.01-2.15)†	1.48 (1.01-2.15)*	3.65 (2.58-5.16)‡	1.28 (0.85-1.92)	2.74 (1.81-4.15)‡
Being indifferent to consequences	1.76 (1.22-2.54)†	1.30 (0.96-1.78)	2.42 (1.83-3.18)‡	0.84 (0.61-1.15)	1.91 (1.38-2.63)‡
Eating more than you should	1.53 (0.90-2.59)	2.76 (1.84-4.14)‡	2.94 (2.04-4.25)‡	1.13 (0.72-1.78)	3.66 (2.26-5.95)‡
Consuming soft drinks and refreshments	1.55 (1.02-2.35)*	1.87 (1.34-2.62)‡	1.96 (1.45-2.66)‡	1.02 (0.71-1.46)	2.96 (2.05-4.29)‡
Eating food with oils or grease	1.74 (0.88-3.43)	4.08 (2.46-6.76)‡	4.73 (2.98-7.51)‡	1.75 (0.97-3.16)	2.66 (1.47-4.80)‡
Eating pastries, chocolates, or candy	2.69 (1.52-4.74)‡	2.49 (1.68-3.70)‡	2.84 (1.98-4.08)‡	1.55 (0.99-2.43)	2.01 (1.30-3.12)†
Eating potato chips, pork-skins or fritters	2.36 (1.19-4.69)*	4.06 (2.53-6.51)‡	3.56 (2.33-5.48)‡	1.47 (0.85-2.52)	2.54 (1.47-4.40)†
Having an unhealthy lifestyle	2.63 (1.67-4.14)‡	1.59 (1.13-2.24)†	2.56 (1.88-3.49)‡	1.13 (0.78-1.62)	1.90 (1.32-2.72)‡
Stress	1.78 (1.33-2.39)‡	0.84 (0.64-1.10)	0.92 (0.73-1.18)	1.12 (0.86-1.47)	1.64 (1.25-2.14)‡
Consequences					
Anxiety	1.37 (0.99-1.89)	1.20 (0.90-1.60)	1.58 (1.22-2.05)‡	0.93 (0.69-1.25)	2.11 (1.57-2.84)‡
Low self-esteem	1.68 (1.22-2.31)‡	1.06 (0.80-1.41)	1.66 (1.29-2.13)‡	0.94 (0.71-1.25)	1.60 (1.21-2.14)‡
Depression	1.61 (1.18-2.19)†	1.12 (0.85-1.48)	1.51 (1.18-1.94)‡	1.03 (0.78-1.36)	1.28 (0.97-1.69)
Feeling tired	1.60 (1.20-2.15)†	0.80 (0.61-1.05)	1.34 (1.05-1.71)*	1.10 (0.84-1.44)	1.35 (1.03-1.77)*
Discrimination	2.46 (1.57-3.87)‡	1.84 (1.30-2.61)‡	2.94 (2.14-4.03)‡	1.57 (1.07-2.29)*	1.20 (0.83-1.73)
Sicknesses	1.88 (0.98-3.61)	2.77 (1.69-4.54)‡	8.36 (5.07-13.79)‡	0.96 (0.56-1.66)‡	3.36 (1.87-6.03)‡
Death	1.82 (1.28-2.60)‡	1.63 (1.21-2.20)‡	1.57 (1.20-2.06)‡	1.26 (0.92-1.72)	1.41 (1.03-1.92)*
Solutions					
Watch less TV	1.57 (1.17-2.12)†	1.00 (0.76-1.31)	1.06 (0.83-1.36)	0.76 (0.58-1.00)	1.85 (1.41-2.43)‡
Spend less time using the PC	1.49 (1.11-1.95)†	0.94 (0.72-1.23)	1.14 (0.89-1.44)	0.74 (0.57-0.96)*	1.47 (1.13-1.92)†
Eating at home	1.87 (1.22-2.86)†	2.11 (1.50-2.96)‡	2.57 (1.89-3.50)‡	1.20 (0.83-1.73)	1.53 (1.06-2.20)*
Personal motivation	2.22 (1.09-4.51)*	3.11 (1.86-5.21)‡	7.94 (4.74-13.30)‡	1.89 (1.03-3.44)*	1.96 (1.09-3.51)*
Treatment with a specialist	2.57 (1.35-4.87)†	3.20 (2.03-5.05)‡	4.87 (3.18-7.44)‡	2.32 (1.34-4.02)†	1.26 (0.76-2.09)
Liposuction	1.00 (0.68-1.49)	0.47 (0.33-0.66)‡	0.30 (0.22-0.41)‡	0.62 (0.43-0.90)*	0.76 (0.53-1.10)
Surgery	0.75 (0.49-1.15)	0.41 (0.29-0.58)‡	0.29 (0.21-0.40)‡	0.60 (0.40-0.89)*	0.91 (0.62-1.34)

*p<0.05;

†p<0.01;

‡p<0.001;

CI=Confidence interval;

OR=Odds ratio

It has been suggested that the readiness to make behavioural changes is preceded by knowledge about the causes and consequences of a disease (13,14), which indicates the importance of exploring and examining the beliefs about the causes of obesity held by populations with high prevalence of diseases (4,11,12,14,15).

According to Hurley *et al*. (14), individuals mostly believe that any behavioural change made will result in benefits that are equal to or higher than the efforts made. Therefore, knowledge about the causes and consequences is not sufficient to implement a behavioural change. Several authors have proposed that behavioural changes depend on different contingencies where family, community, sociocultural environment and policies are important (16,17).

The main strength of this study is the high reproducibility and internal consistency of the questionnaire. It has also been applied to high-risk populations living in two different regions of the Mexico-USA border and was conducted during a six-month period. In addition, this is the first study to our knowledge that explores beliefs about obesity among low-income women living in the Mexico-USA border. Women were chosen as subjects because, in Mexico, the prevalence of obesity is higher in women than in men (18), and women usually play a very important role in children's feeding practices and education (1).

### Limitations

This is a cross-sectional study; the sample was neither representative of the entire population nor did it include all levels of education or socioeconomic groups, and all cultural and ethnic groups within Mexico. Mexico is a large country with a multicultural and multiethnic population. Thus, it is difficult to reach a conclusion without further studies to assess the beliefs of women toward obesity in different regions within the states and ethnic groups. Additionally, in the sets of questionnaire used, prenatal and postnatal causes were not included (19,20); environmental factors (16,17), and treatment efficacy were also absent (21,22). The association of knowledge about causes and consequences of and treatment for obesity with food consumption and the environment was not assessed either.

### Conclusions

The low-income women from two cities in the Mexico-USA border had a reasonably good knowledge about causes and consequences of and treatment for obesity. Nevertheless, there is a significant group that disregarded an unhealthy diet or sedentary lifestyle as causes of obesity. This group is characterized by being less educated, with a normal weight, being younger, and single; it is this high-risk group that needs to be the focus of future prevention policies.

Further studies are warranted to include environmental factors from early to adult life and to identify how the knowledge about causes and consequences of obesity is related to behavioural and attitudinal changes that facilitate modifications at the individual, community, and school level. Those changes might facilitate attitudes that promote policy changes to elicit a less obesigenic environment.

## ACKNOWLEDGEMENTS

We thank Mrs. Martha Estrada-Grimaldo and Adria B. Jimenez for their assistance and editing of the manuscript.
